# Normobaric Hyperoxia Does Not Change Optical Scattering or Pathlength but Does Increase Oxidised Cytochrome *c* Oxidase Concentration in Patients with Brain Injury

**DOI:** 10.1007/978-1-4614-4989-8_10

**Published:** 2012-07-21

**Authors:** Arnab Ghosh, Ilias Tachtsidis, Christina Kolyva, David Highton, Clare Elwell, Martin Smith

**Affiliations:** 1 Institute of Neurology, University College London, 4 Brookfield Road, Queen Square, London, E9 5AH UK; 2 Neurocritical Care, University College Hospitals, Queen Square, London, UK; 3 Medical Physics and Bioengineering, University College London, Malet Place, London, UK

**Keywords:** Hyperoxia, Optical scattering

## Abstract

We report the use of a novel hybrid near-infrared spectrometer for the measurement of optical scattering, pathlength and chromophore concentration in critically ill patients with brain injury. Ten mechanically ventilated patients with acute brain injury were studied. In addition to standard neurointensive care monitoring, middle cerebral artery flow velocity, brain lactate–pyruvate ratio (LPR) and brain tissue oxygen tension were monitored. The patients were subjected to graded normobaric hyperoxia (NBH), with the inspired fraction of oxygen increased from baseline to 60% then 100%. NBH induced significant changes in the concentrations of oxyhaemoglobin, deoxyhaemoglobin and oxidised–reduced cytochrome *c* oxidase; these were accompanied by a corresponding reduction in brain LPR and increase in brain tissue oxygen tension. No significant change in optical scattering or pathlength was observed. These results suggest that the measurement of chromophore concentration in the injured brain is not confounded by changes in optical scattering or pathlength and that NBH induces an increase in cerebral aerobic metabolism.

## Introduction

The identification and avoidance of cerebral hypoxia/ischaemia is a central tenet of contemporary neurocritical care, and near-infrared spectroscopy (NIRS) has a potential monitoring role in this regard. However, whilst a plethora of studies describe the use of “cerebral oximeter” devices—which express absolute scaled concentrations of oxyhaemoglobin and deoxyhaemoglobin ([HbO_2_] and [HHb], respectively) in the form of a combined regional haemoglobin oxygen saturation—few studies outside the context of cardiopulmonary bypass link brain desaturation with neurological outcome or support the routine clinical use of such devices [1].

Cytochrome *c* oxidase (CCO) is the terminal electron acceptor in the mitochondrial electron transport chain and responsible for 95% of cellular oxygen utilisation. Like haemoglobin, the oxidised and reduced forms of CCO have characteristic absorption spectra within the NIR band; these can be measured as the difference spectrum of oxidised–reduced CCO. However, changes in optical scattering and pathlength could potentially confound the accurate measurement of oxidised–reduced CCO concentration ([oxCCO]) in vivo [2].

The aim of this study is to measure optical scattering, pathlength and changes in chromophore concentration in a cohort of brain-injured patients during normobaric hyperoxia (NBH).

## Methods

After approval by the institutional Research Ethics Committee and representative consent, recordings were carried out in ten sedated, mechanically ventilated acute brain-injured patients on the neurocritical care unit. These patients were subjected to NBH protocol, which consisted of a 60-min epoch of baseline recording, followed by 60-min epochs where the inspired fraction of oxygen (FiO_2_) was increased to 60% and then 100%, followed by a final 30-min epoch where FiO_2_ was returned to baseline values. In patients with a baseline FiO_2_ of ≥50%, the 60% FiO_2_ epoch was omitted.

Systemic physiological monitoring included arterial blood pressure (ABP), pulse oximetry and intermittent measurement of arterial blood gases (ABGs)—including partial pressures of oxygen and carbon dioxide (PaO_2_ and PaCO_2_, respectively). Cerebral monitoring was positioned ipsilaterally to the more injured hemisphere and included transcranial Doppler (TCD) ultrasonography measurement of middle cerebral artery flow velocity (Vmca) (DWL Doppler Box, Compumedics Germany), measurement of the lactate–pyruvate ratio (LPR) by cerebral microdialysis (Dipylon Medical AB, Solna, Sweden) and continuous measurement of brain tissue oxygen tension (pbrO_2_) (Licox, Integra Neurosciences, Plainsboro, USA).

The hybrid optical spectrometer has been described in detail elsewhere [3]. Briefly, it comprises two identical broadband spectrometers and a two-channel frequency domain (FD) spectrometer capable of absolute measurements of optical absorption and scattering coefficients (*μ*_a_ and *μ*_s_, respectively) at 690, 750, 790 and 850 nm. [HHb], [HbO_2_] and [oxCCO] were calculated using the UCLn algorithm [4], fitting to changes in NIR attenuation from 780 to 900 nm. Differential pathlength factor (DPF) was derived from *μ*_a_ and *μ*_s_ measured at 790 nm during the baseline period of recording, with additional correction applied for the wavelength dependence of pathlength [5]. Optodes were placed in the mid-pupillary line on the forehead, ipsilateral to the TCD and invasive monitoring. Chromophore concentrations derived from the 35 mm source–detector separation are presented.

Cerebral LPR was measured at 15-min intervals and ABGs at 30-min intervals. The remaining systemic, cerebral and NIRS variables were synchronised and monitored continuously, with the mean value from a noise-free window comprising ≥50% of each entire epoch used for subsequent analysis and reporting.

All values are reported as median  ±  interquartile range (IQR) except where otherwise stated. Probability was calculated using a Wilcoxon signed-rank test comparing the difference between the initial baseline and subsequent study epochs. A Bonferroni correction for repeated measures was applied and *p*  <  0.017 defined as the level of statistical significance.

## Results

Patient demographics are summarised in Table 10.1. The NBH protocol induced significant changes in paO_2_and pbrO_2_, but not in paCO_2_ or Vmca (Table 10.2).

**Table 10.1:** Data on patient demographics

Patient demographics	
Age (range)	45.5 (23–74)
Sex	7 Females, 3 males
Pathology	4 Traumatic brain injury, 6 subarachnoid haemorrhage
Median Admission Glasgow Coma Score (IQR)	7.5 (4–8)

**Table 10.2:** Median (IQR) values for monitored physiological variables during four phases of experiment

	Baseline	FiO_2_ 60%	FiO_2_ 100%	Return to baseline
ABP (mmHg)	89.9 (81.4–95.2)	94.6 (82.9–99.7)	95.2 (90.7–97.1)	90.7 (84.3–93.2)
paO_2_ (kPa)	13.9 (11.7–18.1)	26.6* *(25.8–31.1)*	52.9* *(49.2–57.0)*	11.8 (10.9–14.4)
paCO_2_ (kPa)	4.8 (4.6–5.0)	5.0 (4.8–5)	4.8 (4.6–4.9)	5.1 (4.7–5.5)
pbrO_2_ (kPa)	2.8 (1.6–3.4)	4.0*(2.5–4.7)	7.4*(5.6–8.6)	3.5(1.7–4.7)
Vmca (cm s^−1^)	54.3(51.3–79.6)	56.2(48.7–84.7)	54.3(50.3–83.1)	57.1(51.8–86.1)

Values showing a statistically significant difference from initial baseline are italicised

* *p*  <  0.01

The FD spectrometer failed to collect data at 750 nm in two patients and at 790 and 850 nm in one patient. The remaining data revealed no significant change observed in the values of *μ*_s_ and DPF at any wavelength (Table 10.3).

**Table 10.3:** Median (IQR) values for measured optical scattering coefficient (*μ*_s_) and differential pathlength factors at four wavelengths

	Baseline	FiO_2_ 60%	FiO_2_ 100%	Return to baseline
*μ*_s_ 690 nm (cm^−1^)	10.8(9.05–10.8)	10.6(10.1–12.3)	10.5(9.74–12.4)	10.3(9.39–12.5)
*μ*_s_ 750 nm (cm^−1^)	9.93(9.02–10.2)	10.0(9.04–10.5)	9.76(9.00–10.6)	9.89(8.99–10.7)
*μ*_s_ 790 nm (cm^−1^)	9.48(8.8–9.75)	9.49(8.59–11.5)	9.25(8.25–11.8)	9.10(7.44–11.8)
*μ*_s_ 850 nm (cm^−1^)	9.10(9.0–9.70)	9.40(8.76–9.80)	9.36(8.68–9.54)	9.35(8.9–9.4)
DPF 690 nm	8.40(8.33–8.71)	8.634(8.52–9.44)	8.88(8.49–9.57)	8.54(8.03–9.37)
DPF 750 nm	8.15(7.9–8.24)	8.12(7.76–8.44)	8.32(7.96–9.11)	8.15(7.87–9.05)
DPF 790 nm	8.14(8.12–8.28)	8.24(7.91–8.49)	8.21(7.92–8.81)	7.98(7.89–8.63)
DPF 850 nm	8.07(8.06–8.52)	8.00(7.73–8.40)	7.94(7.66–8.37)	8.00(7.83–8.31)

No variable showed statistically significant variation from baseline

**Fig. 10.1:**
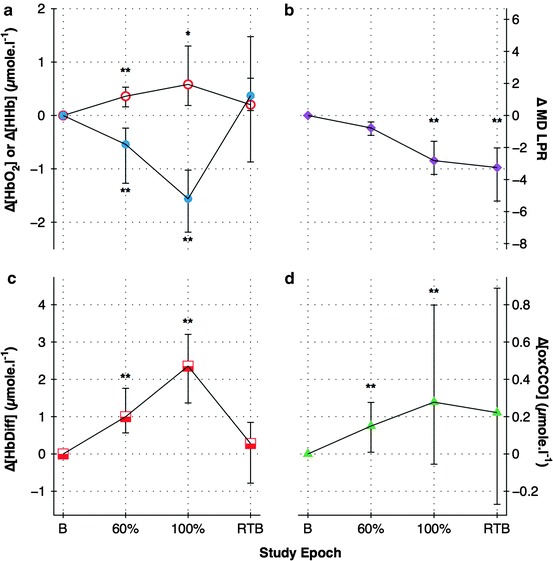
Changes in: (**a**) oxyhaemoglobin (*open circles*) and deoxyhaemoglobin (*solid circles*); (**b**) microdialysate lactate–pyruvate ratio; (**c**) haemoglobin difference; (**d**) oxidised–reduced cytochrome *c* oxidase for each phase of study. *B* baseline, *60%* FiO_2_ 60%, *100%* FiO_2_ 100%, *RTB* return to baseline. *p  <  0.017; **p  < 0.01 comparing study epoch to initial baseline

Changes in chromophore concentration are shown in Fig. 10.1. There was a statistically significant increases in [HbO_2_] and [oxCCO] and decreases in [HHb] during NBH (∆[HbO_2_]: +0.36 and +0.58 μmol l^−1^; ∆[HHb]: −0.54 and −1.56 μmol l^−1^; ∆[oxCCO]: +0.15 and +0.28 μmol l^−1^ during 60% FiO_2_ and 100% FiO_2_ phases, respectively). The baseline median LPR was 24.4 (IQR 22.2–25.7) and showed a statistically significant decrease during NBH (∆LPR: −2.8 and −3.3 during 100% FiO_2_ and return-to-baseline epochs, respectively) and a nonsignificant decrease of −0.8 during the 60% FiO_2_ phase (*p*  < 0.019).

## Discussion

We have used a hybrid optical spectrometer to measure optical scattering, pathlength, changes in oxy- and deoxyhaemoglobin concentrations and changes in CCO oxidation state during NBH in ten critically ill brain-injured patients.

Changes in chromophore concentration, and in particular [oxCCO], have been reported before in a clinical context: the pattern of [oxCCO] change has been related to clinical outcome following cardiopulmonary bypass [6] and our group have previously demonstrated an increase in [oxCCO] with NBH [7] and heterogeneous changes with hypercapnoea [8]. However, changes in optical scattering and pathlength have been proposed as key confounding factors in the measurement of [oxCCO] [2] and the effects of changes in pathlength and scattering on [oxCCO] measurement have been hitherto unknown.

We have adopted a novel approach that combines simultaneous measurement of optical scattering and pathlength in addition to measurement of changes in chromophore concentration and LPR. The concordance between the microdialysis and [oxCCO], combined with the absence of changes in either *μ*_s_ or pathlength, suggests that scattering and pathlength changes do not confound the measurement of chromophore concentration and that the change in [oxCCO] seen during NBH therefore represents an actual increase in mitochondrial aerobic metabolism.

The pattern of chromophore concentration change that we have observed during NBH is similar to that previously reported by our group using different apparatus in a cohort patients who had a similar median age and admission GCS, but had a primary diagnosis of traumatic brain injury rather than the mixed group of traumatic and vascular brain injuries reported in this study. The magnitude of [HbO_2_] increase, however, was smaller in our cohort than that previously reported. The reasons for this are unclear, but may include pathophysiological and instrumentation factors. Interpreting the physiological mechanisms underlying the increase in [oxCCO] in response to NBH is challenging, but may be enhanced by the use of a mathematical model of the cerebral circulation and metabolism [9]. Further work is required to elucidate the relationship between [oxCCO] and changes in cerebral oxygen availability and may aid in the translation of NIRS-derived measurement of [oxCCO] from a research to a clinical tool.
